# Estimating nitrogen and phosphorus concentrations in streams and rivers, within a machine learning framework

**DOI:** 10.1038/s41597-020-0478-7

**Published:** 2020-05-28

**Authors:** Longzhu Q. Shen, Giuseppe Amatulli, Tushar Sethi, Peter Raymond, Sami Domisch

**Affiliations:** 1 University of Cambridge, Department of Zoology, Cambridge, CB2 3EJ UK; 2 Yale University, School of Forestry & Environmental Studies, New Haven, CT 06511 USA; 3 Yale University, Center for Research Computing, New Haven, CT 06511 USA; 4 Spatial-Ecology, Meaderville House, Wheal Buller, Redruth, TR16 6ST UK; 5 Leibniz-Institute of Freshwater Ecology and Inland Fisheries, Department of Ecosystem Research, 12587 Berlin, Germany

**Keywords:** Element cycles, Hydrology

## Abstract

Nitrogen (N) and Phosphorus (P) are essential nutritional elements for life processes in water bodies. However, in excessive quantities, they may represent a significant source of aquatic pollution. Eutrophication has become a widespread issue rising from a chemical nutrient imbalance and is largely attributed to anthropogenic activities. In view of this phenomenon, we present a new geo-dataset to estimate and map the concentrations of N and P in their various chemical forms at a spatial resolution of 30 arc-second (∼1 km) for the conterminous US. The models were built using Random Forest (RF), a machine learning algorithm that regressed the seasonally measured N and P concentrations collected at 62,495 stations across the US streams for the period of 1994–2018 onto a set of 47 in-house built environmental variables that are available at a near-global extent. The seasonal models were validated through internal and external validation procedures and the predictive powers measured by Pearson Coefficients reached approximately 0.66 on average.

## Abstract (summary)

**Table:** 

Measurement(s)	concentration of nitrogen atom in water • phosphorus atom
Technology Type(s)	machine learning
Factor Type(s)	geographic location • seasonal measurement • year of data collection
Sample Characteristic - Environment	stream • river • fresh water body
Sample Characteristic - Location	contiguous United States of America

Machine-accessible metadata file describing the reported data: 10.6084/m9.figshare.11948916

## Background & Summary

Nitrogen (N) and phosphorus (P) are key nutritional elements for many important life processes such as protein and DNA synthesis, primary production, cellular growth and reproduction. Both have a natural global cycle that includes conversion between different inorganic and organic forms, solid and dissolved (and gaseous for nitrogen) phases that maintained their pre-industrial concentrations within certain natural bounds. During the preindustrial era, the concentrations and fluxes of N and P in rivers were generally small, much less than present day levels, and were mainly sourced from erosion and the leakage of dissolved N and P in their organic/inorganic forms^1,2^. However, today anthropogenic production of N and P to support fertilisation and industrial releases^3,4^ has dramatically increased the N and P presence in water bodies. This has led to the widespread eutrophication of both inland and coastal waters^5^.

Over the past decades, significant progress has been made towards our understanding of the dynamics of natural and anthropogenic inputs of N and P to inland waters. Furthermore, the recognition of human impact on the N and P cycle has driven much research into the scope for better management of these nutrients^5,6^. However, our current ability to map N and P concentrations across regions or the globe is still limited. Early attempts focused on concentrations and fluxes from major rivers^3,7^ and were implemented through bottom-up approaches, which estimated N and P content based on our knowledge of land-use and population influences on river nutrients^8–11^. Other local and regional studies have also featured different combinations of bottom-up, process based, and statistical models, which link N concentrations in inland water to environmental variables^12–15^.

Freshwater environmental variables (climate, topography, land cover, surface geology and soil) that account for the basin and upstream environment have recently been computed^16^. This set of stream variables at the near-global scale provides a new base for stream-relevant biotic and abiotic modelling, such as variability in biodiversity, nutrient distributions, or water flows. Based on this platform, we present a new method for mapping the concentrations of N and P in various chemical forms across continental waters based on a machine learning approach. The resulting N and P maps can be used to study nutrient loading and processing in inland waters. For instance, fertiliser run-off presents a high load of chemical nutrients in recipient freshwater bodies, and can be charted by the aforementioned method^17,18^. The N and P maps possess information about the location of nutrient-enriched streams, which can guide engineered de-nitrification processes^19,20^. In addition to resource recovery, a mitigation strategy can be employed through the improved management of nutrient-rich wastes. In this approach as well, the derived N/P ratio map can prove a valuable source of information on where N vs P limitation might be located regionally. Furthermore, this unique N and P modelling can be used in conjunction with process-based methods to enhance the understanding of metabolism and recycle of N and P in riverine systems.

In this paper, we present a gridded geo-dataset^21^ (in form of GeoTIFF raster layers) derived by connecting freshwater environmental variables with *in situ* measurements and map the distribution of various N and P compounds in water bodies across the conterminous US for the period of 1994–2018 recorded in the Water Quality Portal (WQP)^22^. Random Forest (RF)^23^, a well-established machine learning algorithm was employed in this study for its exceptional capability of handling complex and heterogeneous data. We demonstrate in detail below how RF has excelled to date at capturing local geographical variations of stream predictors, and produces superior predictability for N and P distributions in the US. The mapped resolution of the predicted N and P concentrations is at a 30 arc-second (∼1 km) gridded stream network^16,24^ for four seasons. Moreover, the quality and appeal of the proposed geo-dataset^21^ lies in the rigorous scripting and modelling procedures that was applied to treat sparse spatio-temporal observations. Additionally, the computation was performed by employing multi-core processing in a super computer which requires advanced geocomputation programming skills. The described geo-dataset^21^ is ready for use as input data in various environmental models and analyses. The newly developed geo-dataset^21^ and the methodological framework are suitable for large-scale environmental analyses such as N and P emissions in small and large rivers at a global scale. To our knowledge, this is the first time that N and P concentrations have been estimated at such high spatial resolution for the territory large as the contiguous US.

## Methods

The Methods section is divided into two subsections that includes: (i) Data pre-processing, that describes cleaning the gauge stations source data (measured N and P concentrations, referred hereafter as observations or response variables), spatial/seasonal variability and stream layers (referred hereafter as predictors); (ii) Modelling framework, that concerns data splitting and model training/validation/prediction.

### Source datasets and pre-processing

#### N and P concentration data source–observations

The U.S. Geo-logical Survey (USGS), the U.S. Environmental Protection Agency (EPA) and the National Water Quality Monitoring Council developed the Water Quality Portal (WQP)^22^, which is so far the largest standardised water quality database^25^. From WQP^22^, we retrieved the measured concentration data for N and P nutrients in their various chemical forms for the period from 1994 to 2018 with data spanning US stream networks. Each single observation is associated with its sampling geolocation (latitude and longitude) and a USGS Parameter Code (PC) to indicate its chemical identity. We selected five nutrients (referred to as “chemical species”) of interest as the response variables (see Table 1).

**Table 1:** Chemical nutrients with their USGS Parameter Code (PC) and abbreviation.

PC	Description	Abbreviation
00600	Total Nitrogen	TN
00665	Total Phosphorus	TP
00602	Total Dissolved Nitrogen	TDN
00666	Total Dissolved Phosphorus	TDP
00618	Nitrate	NO3

#### Data transformation and cleaning

The chemical nutrients recorded in WQP^22^ were provided by multiple organisations^26^. Employing such multi-sourced data for the “secondary use”, i.e. beyond the original intention proposed by the original data collection agencies^26^, can result in a number of challenges. For instance, intermittent sampling activities and data gaps in time series complicated the temporal analyses for long-term trend. Data records can be misinformative owing to instrument failure, missing measurement that are labelled as “0” values and incorrect use of physical units^25,26^. Such errors might produce extreme values beyond the natural value range and trend (for example, hypothetically TN could range from 0.002 to 20.5 ppm while values exceeding 200 ppm are considered unrealistic), and also large number of “0” values). We removed extreme values by data trimming using certain thresholds.

The distribution of the raw observation data at day-level resolution for all nutrients (TN, TP, TDP, TDP, and NO3) were highly left-skewed, as quantified by the third standardised moment (Eq. 1)

1 $${\widetilde{\mu }}_{3}=E\left[{\left(\frac{X-\mu }{\sigma }\right)}^{3}\right]$$

where *μ* is the mean and *σ* is the standard deviation and *E* is the expected value.

We reported the computed skewness values on the plots in the Supplementary Figures 1 and 2. We then applied the Box–Cox power transformation^27^ (Eq. 2) on the raw data to improve their symmetry (see better linear behaviour in the Q-Q plots). Assuming the transformed data are nearly normal distributed, we retained only the data within the non-rejection zone at the *α* level of 0.05 to reduce the influence of extreme values.

2 $${y}^{\lambda }=\left\{\begin{array}{cc}\frac{{y}^{\lambda }-1}{\lambda }, & if\,\lambda \ne 0,\\ {\rm{\log }}(y), & if\,\lambda =0.\end{array}\right.$$

As another layer of data filtering, we retrieved and retained only the data with the number of observations equal or greater than three in a single month, and with a Coefficient of Variation (CV) less than two, determined by iterative trials. The data after cleaning are reported in Table 2 and were further used for the analysis.

**Table 2:** Number of observations of the nutrients for each of the four seasons, remained after the data cleaning.

Season	Winter	Spring	Summer	Autumn
Month	11-12-01	02-03-04	05-06-07	08-09-10
TN	1651	3090	3220	2254
TDN	678	1158	1237	875
NO3	1628	2761	3314	2238
TP	2595	4831	5860	4155
TDP	911	1651	2175	1412

#### Spatial and seasonal variation

**Fig. 1:**
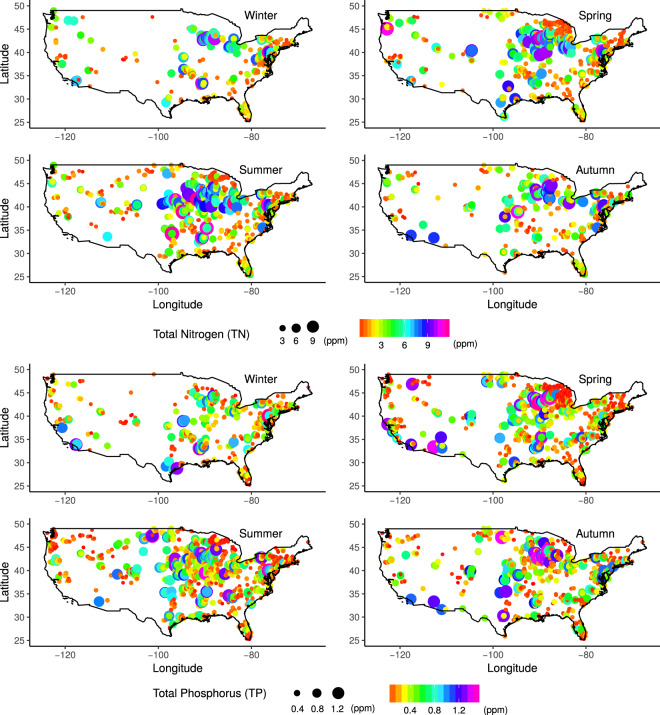
Spatio-temporal distribution of TN and TP. Spatial and seasonal distribution of the Water Quality Portal’s stations. The Total Nitrogen (TN) and Total Phosphorus (TP) seasonal mean for each station is labelled by a colour circle which also increase in size in accordance to the value TN and TP values.

We performed spatial and temporal analyses to better inform the design of modelling strategies. Within the current data set, we identified only eight stations with eight or more years of data continuity for a single chemical species (see Supplementary Fig. 3). The Kwiatkowski Phillips Schmidt Shin (KPSS) tests^28^ rejected the null hypothesis that a temporal trend exists in the time series. Additionally, we plotted the data distributions across the continuous US for each year in Supplementary Fig. 4. From these plots, we noticed great intra-annual spatial variability in the data and a static trend for the mean through all years. Based on this result, we performed seasonal mean aggregations for the full time period (1994–2018). Furthermore, we investigated the seasonal spatial variability by examining the aggregated seasonal mean across years with observations and plotted the data distributions as shown in Fig. 1 and Supplementary Figures 5–7. For a better visual effect, we computed the seasonal difference maps for each nutrient as shown in Supplementary Fig. 8. The colours in the RGB maps vary based on the differences between two seasons, where white areas indicate greater similarities between each other and black areas indicate no data.

#### Stream layers - predictors

To build the predictive models, we used a total of 47 predictors belonging to four categories: topography^24^, soil^29^, land cover^30^ and climate^31^ (Table 3). All predictors are freshwater-specific environmental variables^16^ that have accounted for the upstream characteristics of the watershed and longitudinal connectivity across the 30 arc-second HydroSHEDS stream network^24^. For each grid-cell on the stream network, the upstream catchment and stream were delineated, i.e., where each grid-cell served as a virtual pour-point overlaid with range-wide environmental layers (Table 3). Subsequently these data were averaged across lakes and reservoirs from the Global Lakes and Reservoir dataset^32^ and smoothed at river in- and outlets^16^. All primary environmental data from the four categories had a native 1 km spatial resolution, and we calculated the upstream average (topography, soil, land cover and temperature) and sum (precipitation) across each sub-catchment. Here, soil data refers to the soil within the depth of 2.5 cm (0–5 cm thickness)^29^. This yielded a series of predictors such as the upstream average forest cover, upstream sum of precipitation that mimics surface run-off and the average upstream temperature^16^, available at www.earthenv.org/streams.

**Table 3:** Stream environmental predictors. Overview of all 47 environmental predictors used in the models.

Variable type	Variable name	Variable description	Variable Alias
elevation	dem	Average elevation	dem_avg
slope	slope	Average slope	slope_ave
topology	ord	Stream order	lentic_lotic01
soil	soil01	Soil organic carbon	soil_avg_01
	soil02	Soil pH in H2O	soil_avg_02
	soil03	Sand content mass fraction	soil_avg_03
	soil04	Silt content mass fraction	soil_avg_04
	soil05	Clay content mass fraction	soil_avg_05
	soil06	Coarse fragments (>2 mm fraction) volumetric	soil_avg_06
	soil07	Cation exchange capacity	soil_avg_07
	soil08	Bulk density of the fine earth fraction	soil_avg_08
	soil09	Depth to bedrock (R horizon) up to maximum 240 cm	soil_avg_09
land cover	soil10	Probability of occurrence (0–100%) of R horizon	soil_avg_10
	lc01	Evergreen/deciduous needleleaf trees	lu_avg_01
	lc02	Evergreen broadleaf trees	lu_avg_02
	lc03	Deciduous broadleaf trees	lu_avg_03
	lc04	Mixed/other trees	lu_avg_04
	lc05	Shrubs	lu_avg_05
	lc06	Herbaceous vegetation	lu_avg_06
	lc07	Cultivated and managed vegetation	lu_avg_07
	lc08	Regularly flooded shrub/herbaceous vegetation	lu_avg_08
	lc09	Urban/built-up	lu_avg_09
	lc10	Snow/ice	lu_avg_10
	lc11	Barren lands/sparse vegetation	lu_avg_11
	lc12	Open water	lu_avg_12
temperature	tmin	Monthly temperature average min	
temperature	tmax	Monthly temperature average max	
precipitation	prec	Sum of monthly precipitation	
	hydro01	Annual Mean Upstream Temperature	hydro_ave_01
	hydro02	Mean Upstream Diurnal Range (Mean of monthly (max temp - min temp))	hydro_ave_02
	hydro03	Upstream Isothermality (hydro02 / hydro07) (* 100)	hydro_ave_03
	hydro04	Upstream Temperature Seasonality (standard deviation *100)	hydro_ave_04
	hydro05	Maximum Upstream Temperature of Warmest Month	hydro_ave_05
	hydro06	Minimum Upstream Temperature of Coldest Month	hydro_ave_06
	hydro07	Upstream Temperature Annual Range (hydro05 - hydro06)	hydro_ave_07
	hydro08	Mean Upstream Temperature of Wettest Quarter	hydro_ave_08
	hydro09	Mean Upstream Temperature of Driest Quarter	hydro_ave_09
hydroclimate	hydro10	Mean Upstream Temperature of Warmest Quarter	hydro_ave_10
	hydro11	Mean Upstream Temperature of Coldest Quarter	hydro_ave_11
	hydro12	Annual Upstream Precipitation	hydro_ave_12
	hydro13	Upstream Precipitation of Wettest Month	hydro_ave_13
	hydro14	Upstream Precipitation of Driest Month	hydro_ave_14
	hydro15	Upstream Precipitation Seasonality (Coefficient of Variation)	hydro_ave_15
	hydro16	Upstream Precipitation of Wettest Quarter	hydro_ave_16
	hydro17	Upstream Precipitation of Driest Quarter	hydro_ave_17
	hydro18	Upstream Precipitation of Warmest Quarter	hydro_ave_18
	hydro19	Upstream Precipitation of Coldest Quarter	hydro_ave_19

All predictors except for climate were static, as opposed to being time-updated. Monthly climate data was averaged to a seasonal level as described in Table 2. Regarding the temperature layers, we only aggregated the upstream air temperature across the stream cells within the sub-catchment, while all other layers were aggregated across the entire sub-catchment area^16^. The unit for each stream variable is derived from an original, spatially continuous environmental variable across the land surface area. Thus, temperature is expressed in degrees Celsius, precipitation in millimetres, and land cover as a percentage of each class (e.g. Urban/built-up class in percentage). We refer to^16^ for further details regarding the calculation of the freshwater-specific predictors.

#### Snapping gauge station locations to the stream network

Due to the possible spatial discrepancy between the HydroSHEDS stream network and the gauge station locations, the latitude and longitude locations of the gauge stations do not consistently fall directly on the stream grids. Hence, we snapped the geolocations (latitude and longitude) of the stations to the HydroSHEDS stream network using the *r*.*stream*.*snap* function in GRASS GIS^33^ with 3 km as the maximum distance tolerance. After snapping, we computed the seasonal mean for each chemical species by considering all the points that fell in the same snapped location. This led to a unique one-to-one association between a geographical identification and an averaged concentration value for each season and each chemical species.

### Modelling framework

#### Data splitting procedure

We split the full dataset into two sub-datasets, training and testing respectively. To consider the heterogeneity of the spatial distribution of the gauge stations, we employed the spatial density estimation technique in the data splitting step by building a density surface using Gaussian kernels with a bandwidth of 50 km (using *v*.*kernel* available in GRASS GIS^33^) for each species and season. The pixel values of the resultant density surface were used as weighting factors to split the data into training and testing subsets that possess identical spatial distributions.

In order to optimise the split ratio between the training and testing subsets, we explored the Mean Root Square Error (MRSE = $\sqrt{{\sum }_{i}^{n}{({x}_{i}-{\widehat{x}}_{i})}^{2}}/n$, where *x*_*i*_ represents the observation and ${\widehat{x}}_{i}$ represents the predicted value for data (*i*) at various proportions of the training-testing subsets (60–40%, 70–30%, 80–20%, 90–10%) with 50 times independent samplings for each trial. The trial repetition intended to sample different combinations of training and testing so as to reduce the bias of the sample estimate. To this end, we labelled the MRSE as ${{\rm{MRSE}}}_{te}^{or}$ for the testing sub-dataset in its original values (ppm) and ${{\rm{MRSE}}}_{te}^{bc}$ for the testing sub-dataset in its Box-Cox transformed values.

As shown in the Supplementary Fig. 9, we noticed a monotonic increase of the ${{\rm{MRSE}}}_{te}^{bc}$ and ${{\rm{MRSE}}}_{te}^{or}$ for all models as the splitting ratio increased from 0.5 to 0.9. Given the consistent low MRSE_*te*_ and its low variability (defined as the standard deviation of MRSE_*te*_) at the proportion 0.5, we decided to use it as the optimal cut to build the final models.

#### Model training

We employed the RF regression algorithm implemented in the R-package *randomForestSRC*^34,35^ to train the models. RF regression is an ensemble learning strategy that elevates the collective predictive performance of a large group of weaker learners (regression trees). Two key elements contributing to the superiority of the RF algorithm are bootstrapping aggregation (bagging) and random selection of variables. Bagging (bootstrap sampling from the training sub-dataset) aims at reducing data noise through averaging. Data that is not included in the bag is called an out-of-bag (OOB) sample. Random drawing of variables improves variance reduction by reducing the intercorrelation between trees. OOB samples can be used to validate the model performance (equivalent to cross validation) and evaluate the variable importance. The variable importance is of great value in identifying the most influential variables that direct predictive outcomes and thus offer adaptive or intervention strategies in response to the modelled phenomena. One important feature of the RF algorithm is its relative resilience towards data noise due to the two mechanisms mentioned above. This technical advantage of RF directly benefits the analysis of environmental data. The attractiveness of the *randomForestSRC* package was that it allows considering the sample distribution density in the bagging step. In the model development, we paid close attention to the model stability. We noticed that the superparameter as the number of trees had a strong impact on the model errors as shown in Supplementary Fig. 10. In the end, we used 6000 trees for each model as all models achieved stabilisation by approaching this number.

#### Model validation

The predicting performance on the training and testing sets provided complementary information for the model validation. Training primarily exhibits model robustness, i.e. stability and balance of model predictability in the presence of data shuffling. Testing measures the model performance on the unseen data and addresses the model fitness. In this context we used the Pearson correlation coefficient as the statistical metric to quantify the predictive performance of the models.

To supplement the Pearson correlation coefficient and provide an in-depth assessment of model accuracy, we calculated the Root Mean Square Error (RMSE = $\sqrt{{\sum }_{i}^{n}{({x}_{i}-{\widehat{x}}_{i})}^{2}/n}$, where *x*_*i*_ represents the observation and ${\widehat{x}}_{i}$ represents the predicted value for data *i*) to numerically quantify model uncertainty, since the it offers a more discernible measure of prediction accuracy. Thus, we denote:

(i) ${{\rm{RMSE}}}_{te}^{or}$ and ${{\rm{RMSE}}}_{tr}^{or}$ for the testing/training sub-dataset in their original physical unit (ppm); (ii) ${{\rm{RMSE}}}_{te}^{bc}$ and ${{\rm{RMSE}}}_{tr}^{bc}$ for the testing/training sub-dataset for their Box-Cox transformed values.

RMSE can also be used to obtain a comparison of accuracy across high and low-density gauge station distribution. To this end, we calculated a partial ${{\rm{RMSE}}}_{te}^{or}$ by sorting the sub-datasets in accordance with density surface values, and referring to points below the 20^th^ and above the 80^th^ percentiles, obtaining ${{\rm{RMSE}}}_{te.ld}^{or}$ and ${{\rm{RMSE}}}_{te.hd}^{or}$ respectively. Finally, to illustrate the geographic distribution of these errors we plotted the residual maps for the conterminous US.

Lastly, after establishment of the predictive models, we investigated the contributions of each variable to the predicted outcomes by means of the “variable importance”, an output from RF.

#### Model prediction

The final validated RF models were applied to predict each of the 30-arc-second stream grid cell within the conterminous US, for all the nutrients (TN, TP, TDP, TDP and NO3). The predictive outcomes were then reversely transformed back to recover their original physical values (in ppm).

## Data Records

We provide TN, TDN, NO3, TP, and TDP concentrations (ppm) for four seasons (winter, spring, summer and autumn) for the gridded stream network at a spatial grain of 30 arc-second (∼1 km). All layers are available for download at PANGAEA repository^21^. The nutrient concentrations, mapped across the conterminous USA, are available in a compressed GeoTiff file format in the WGS84 coordinate reference system (EPSG:4326 code). All layers are stored as floating points (Float32 data type) to ensure sufficient precision for future use and analysis for varied purposes.

The predicted nutrient maps follow the layer name convention:

nutrient abbreviation_resolution_season.format

Below are two examples of the layer names for the two main nutrients product TN and TP

- TN_1KM_winter.tif: layer showing the Total Nitrogen for the winter season at 30 arc-second spatial resolution.
- TP_1KM_summer.tif: layer showing the Total Phosphorus for the summer season at 30 arc-second spatial resolution.

**Fig. 2:**
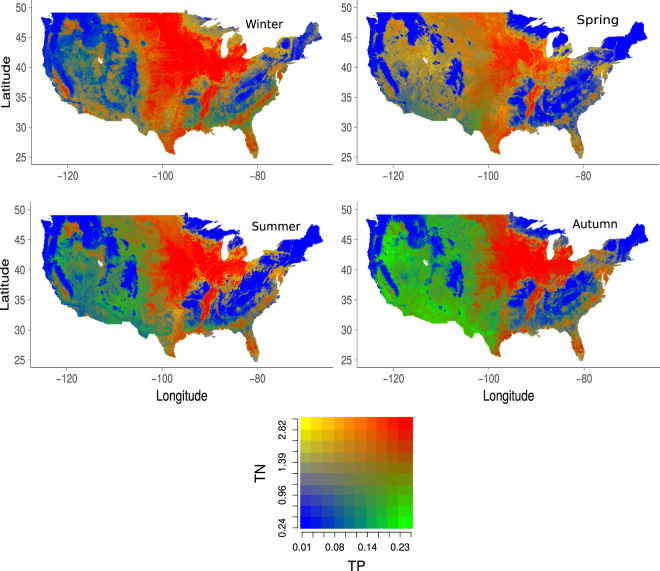
Bivariate maps for TN and TP. Bivariate maps showing the predicted Total Nitrogen (TN) and Total Phosphorus (TP) values in ppm across the four seasons. Streams and rivers on the original 30 arc-second resolution maps were aggregated using the mean value of a moving window with 10 × 10 grid-cells for an improved visualisation. Red indicates high concentration areas, which mainly coincide with high agriculture or grazing activities or urban zones. Blue indicates low nutrient load areas, which are frequently occupied by forests or deserts.

For the purpose of visual interpretation of the results, we plotted the TN and TP bivariate maps as shown in Fig. 2 and Supplementary Figures 11 and 12. The bivariate TN-TP map representation permits an immediate perception of the spatial patterns of these two nutrients in the same map. This visual result was achieved by a mean-value aggregation of the original 30 arc-second resolution nutrient distributions using a moving window of 10 × 10 grid-cells so that a continuous surface could be easily mapped across the entire conterminous US. Figure 2 shows high concentrations of TN and TP (red colour) in intensive agriculture/grazing areas (e.g. of the US Midwest) and also close to large urban areas (e.g. New York, Philadelphia, Baltimore, Washington DC). On the other hand, low concentrations of TN and TP are located in forestry/mountain areas (e.g. Rocky Mountains, Appalachian Mountains). This observation is in line with the anthropogenic eutrophication effect that coincides with intensive agricultural activities and urban waste water^36^.

## Technical Validation

**Fig. 3:**
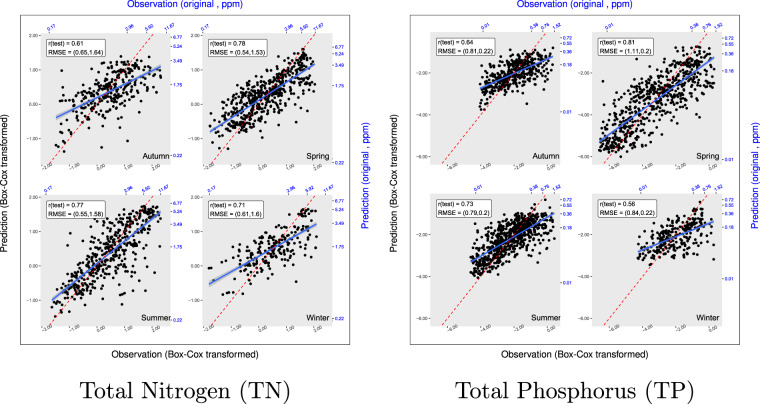
Correlation plots for TN and TP in testing. Seasonal correlation plots for TN and TP for the testing data sets. Horizontal axes represent the observations and vertical axes represent the predicted values. Ticks labelled in black are box-cox transformed values and ticks in blue are original values in ppm. Pearson coefficients (r) and RMSE(${{\rm{RMSE}}}_{te}^{bc}$, ${{\rm{RMSE}}}_{te}^{or}$) are given in the upper-left corner box.

The Pearson correlations between predicted and observed values for TN and TP are in the range of 0.56–0.81 across the testing sets as shown in Fig. 3. The red dotted lines represent the 1:1 relationship for each panel. The solid blues lines showed the regression of the black data points (predictions vs observations). Similar plots were generated for TDN, TDP and NO3 (see Supplementary Fig. 13). The high-level correlation for each plot and overall consistency among all species suggested the appropriate fitting for all models. The correlation graphs for the training set (TN, TP,TDN, TDP and NO3) are provided in the Supplementary Fig. 14.

**Fig. 4:**
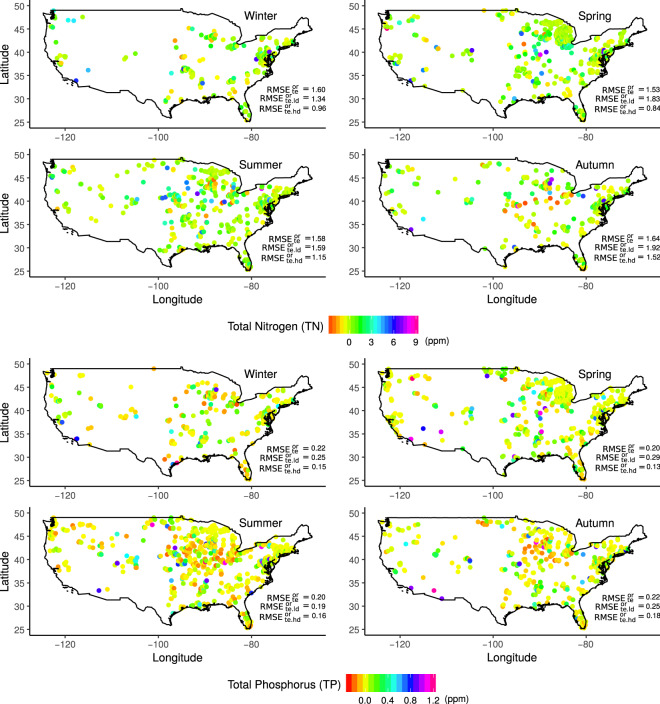
Residual maps for TN and TP. Residuals are computed using the testing sub-dataset (observations minus predictions). In each maps is also reported the ${{\rm{RMSE}}}_{te}^{or}$ for the testing sub-daset in ppm, ${{\rm{RMSE}}}_{te.ld}^{or}$ and ${{\rm{RMSE}}}_{te.hd}^{or}$ using observation in the low/high density, respectively.

In Fig. 4 and Supplementary Fig. 15 we mapped the residual (observation minus prediction) of the testing sub-dataset across the conterminous US. We also reported the overall ${{\rm{RMSE}}}_{te}^{or}$ and RMSEs in areas with low (${{\rm{RMSE}}}_{te.ld}^{or}$) and high (${{\rm{RMSE}}}_{te.hd}^{or}$) station densities. The ${{\rm{RMSE}}}_{te.ld}^{or}$ results slightly higher than the ${{\rm{RMSE}}}_{te.hd}^{or}$, nonetheless they are very close to the overall ${{\rm{RMSE}}}_{te}^{or}$. These results show that the model is able to perform reasonably well also in areas with low presence of gauge stations.

From the residual maps we also noticed that the model sometimes underestimates the higher values. Three possible causes may have contributed to this result: (i) untrustful observations (ii) anthropogenic actions that are not fully included in the current environmental variable layers (such as tile drainage^37^), which highlighted the significance of human influence and suggested the need for further completing the variable list (iii) the original highly skeweness of the observation data and the associated box-cox transformation implemented.

As shown in the Supplementary Figures 16 and 17, all predictors have been ranked according to their relative importance. We noticed that the predictor lu_avg_07 corresponding to the cultivated vegetation played a dominant role for three seasons in the TN prediction. This observation seems logical since nutrient deposition on the cultivated land can run off into nearby streams to influence the local TN concentration. For another example, soil_avg_02 corresponding to pH in soil outweighed all other predictors in the TP prediction for three seasons, referring to the acidic nature of most phosphorus compounds.

## Usage Notes

The newly-developed stream nutrient concentration layers^21^ have a wide array of potential applications in stream ecology, biodiversity research, conservation science, and stream and lake restoration ecology. For instance, the layers can be used to quantify the overall mass of of N and P discharged into a specific lake or ocean body, enabling a deeper understanding of global-scale eutrophication^38^. Furthermore, these statistical estimates of nutrient concentration can be used to verify new process-based models that predict nutrient concentrations and transformations in inland waters worldwide^39^. The estimates can also be combined with maps of soil nutrient levels and fertiliser use to obtain information on terrestrial-aquatic coupling^40,41^. Finally, the stoichiometry of the N/P ratio in natural/ecological systems is vital information for studying metabolic and biogeochemical processes. These new ratio maps can be used to enhance our knowledge on how coupled biogeochemical cycles impact ecosystems^42^.

Overall, the newly-developed layers provide the basis for a variety of high-resolution, nutrient-related analyses across the inland waters in the conterminous US. A global-scale N and P assessment with new stream predictors at higher resolution (3-arc-second) is under development by our group. The focus is on creating new geomorphometry variables (Geomorpho90m^43^) based on MERIT-DEM^44^ by adopting the procedure described in^45^. The MERIT-DEM derived stream network is also under development^46^. These former described layers will be useful in combination with other global maps of irrigated areas^47^, livestock^48^, agricultural fertiliser use^49^, soil types/properties^50^ to compute N and P concentrations more accurately on a global scale. We encourage potential users of the described geo-dataset to contact the authors for future product updates.

## Supplementary information

SUPPLEMENTARY INFORMATION

## Data Availability

We used the following open source software packages to compute the full processing chain: ● Geospatial Data Abstraction Library (GDAL, version number 2.1.2)^51,52^. ● Geographic Resources Analysis Support System software (GRASS, version number 7.4.0)^33,53,54^. ● Processing Kernel for geospatial data (PKTOOLS, version number 2.6.3)^55,56^. ● R: a language and environment for statistical computing^57^, with the following libraries: randomForestSRC^34,35^, geoR^58^, plyr^59,60^, moments^61^, data.table^62^, reshape^63,64^, dplyr^65^, ggplot2^66,67^ All of these tools provide fast and scalable functions for raster-based workflows that are easily automated using a scripting language, such as Bash or Python^68^. They also allow for the processing of very large geo-datasets owing to efficient algorithms and optimised memory management. In the spirit of reproducible research we provide the scripting procedure at the GitLab repository (https://gitlab.com/Ferdinand18/np_us_streams). The full procedure, starting from the N and P observations treatment to the 30-arc-second raster predictions, is provided below. ● 01_Cleaning.sh: cleaning the raw observation data. ● 02_Snapping.sh: snapping the observation data points onto the gridded stream network. ● 03_Extraction.sh: extracting descriptors corresponding to the snapped points. ● 04_Modelling.sh: building predictive models based on the observation data. ● 05_Prediction.sh: making predictions for all the US streams and building gridded GeoTiff maps as the final output.
