# Supplementary material for: Estimating nitrogen and phosphorus concentrations in streams and rivers, within a machine learning framework
Source: Sci Data. 2020 May 28;7:161. doi: 10.1038/s41597-020-0478-7 (PMC7256043; doi:10.1038/s41597-020-0478-7)
Supplement: Supplementary file 1 — SUPPLEMENTARY INFORMATION [file 41597_2020_478_MOESM1_ESM.pdf]

Supplementary Information

List of Figures

1 **Data distribution plots for TN, TDN, and NO3** . . . 1
2 **Data distribution for TP and TDP** . . . 2
3 **Time series plots** . . . 2
4 **Annual trend violin plots** . . . 3
5 **Distribution map for NO3** . . . 4
6 **Distribution map for TDN** . . . 4
7 **Distribution map for TDP** . . . 5
8 **Seasonal variation RGB maps** . . . 6
9 **Splitting variations.** . . . 7
10 **Plots for model tuning** . . . 8
11 **Bivariate maps for TDN and TDP** . . . 9
12 **Seasonal prediction maps for NO3** . . . 10
13 **Correlation plots for TDN, TDP and NO3 in testing** . . . 11
14 **Correlation plots for all chemical species in training** . . . 12
15 **Residual maps for TDN, TDP and NO3** . . . 13
16 **Variable importance plots for TN and TP** . . . 14
17 **Variable importance plots for TDN, TDP and NO3** . . . 15

Note: all the figures in the Supplementary Information are plotted at high resolutions, we suggest to zoom in to see additional details.

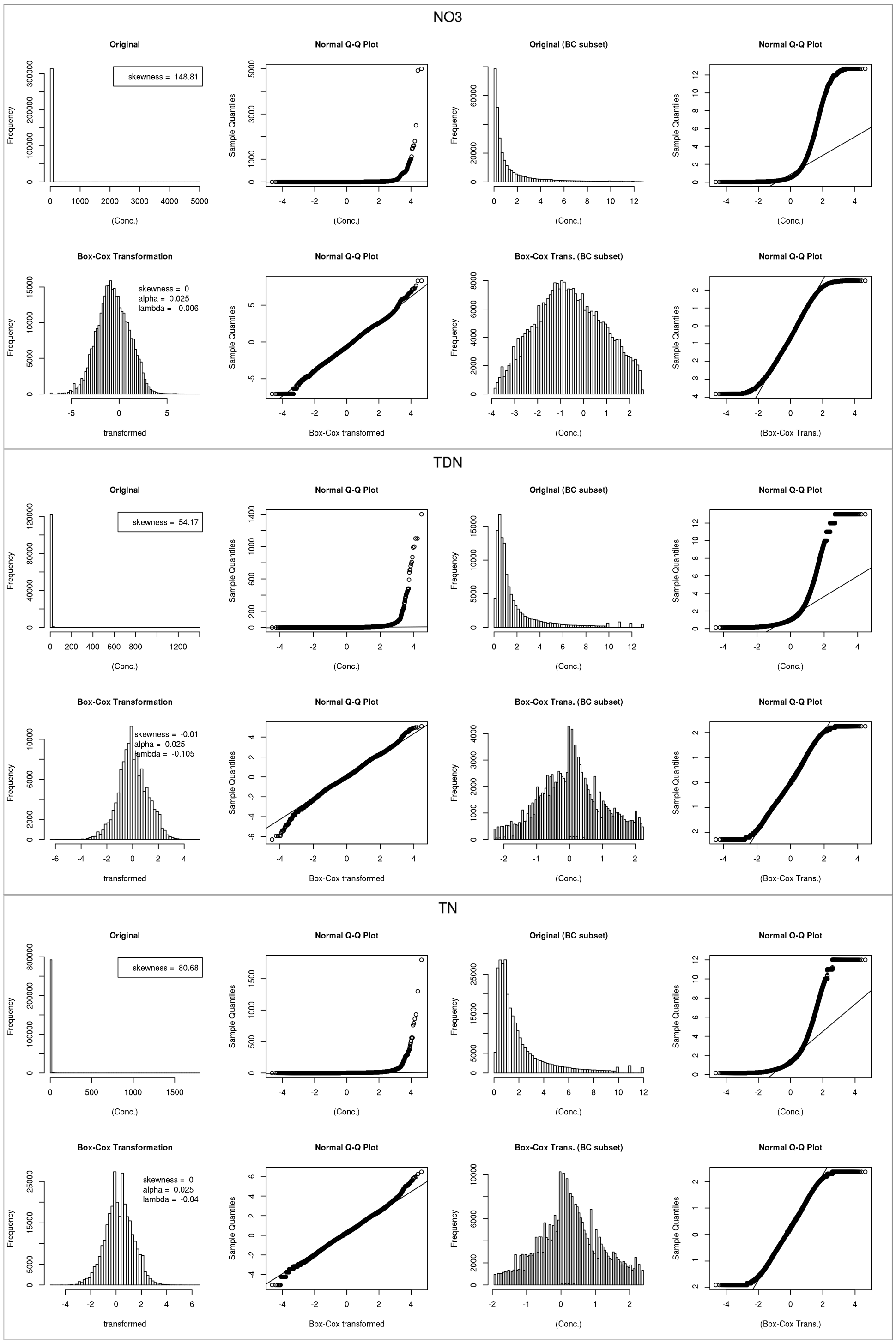

Figure 1: **Data distribution plots for TN, TDN, and NO3.** Column 1 and 2 of each species are the original data. Column 3 and 4 are the subsets after removing the 2.5 and 97.5 percentile of Box-Cox transformed data.

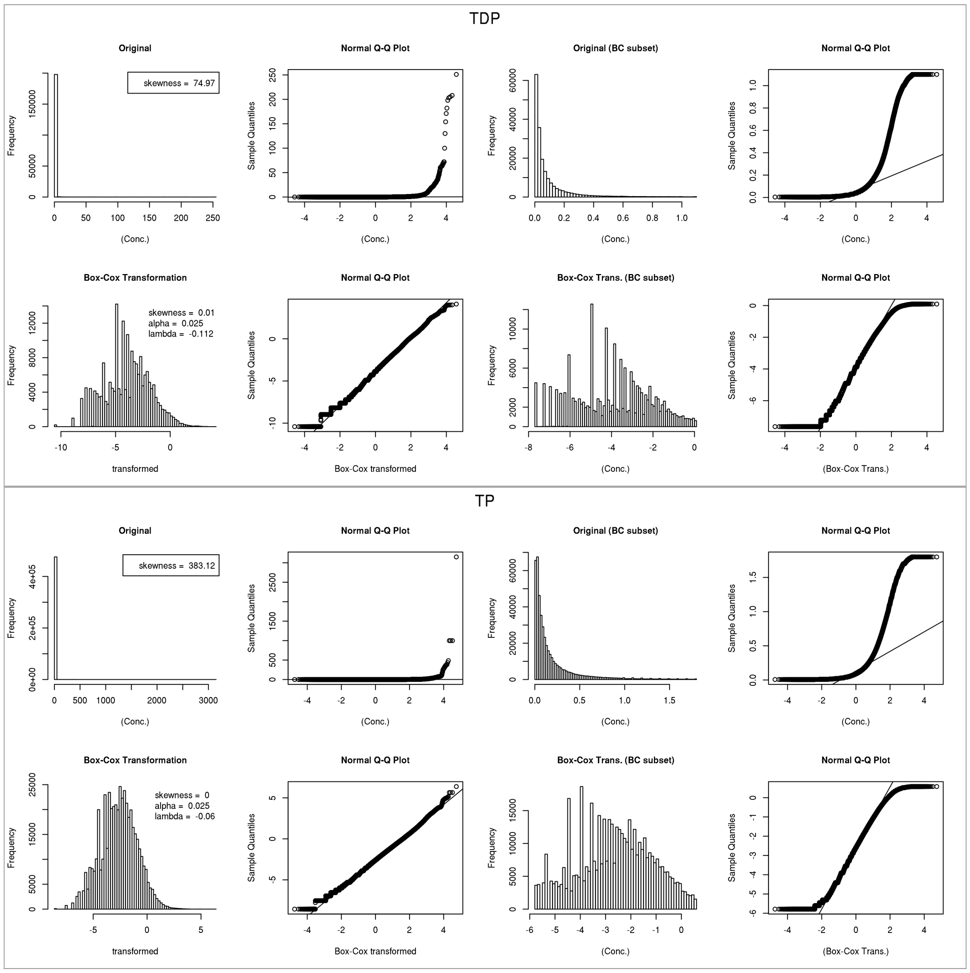

Figure 2: **Data distribution for TP and TDP.** Data distribution plots for TP, TDP. Column 1 and 2 of each species are the original data. Column 3 and 4 are the subsets after removing the 2.5 and 97.5 percentile of Box-Cox transformed data.

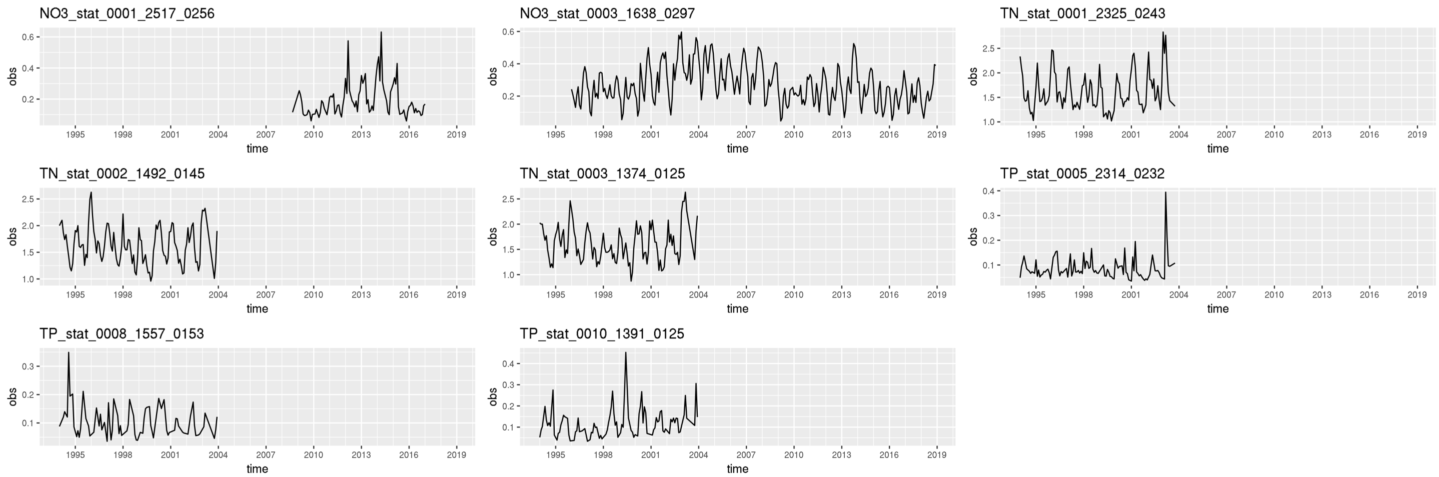

Figure 3: **Time series plots** Time series plots for monthly averaged data of the stations with eight or more than eight years of continuous observations.

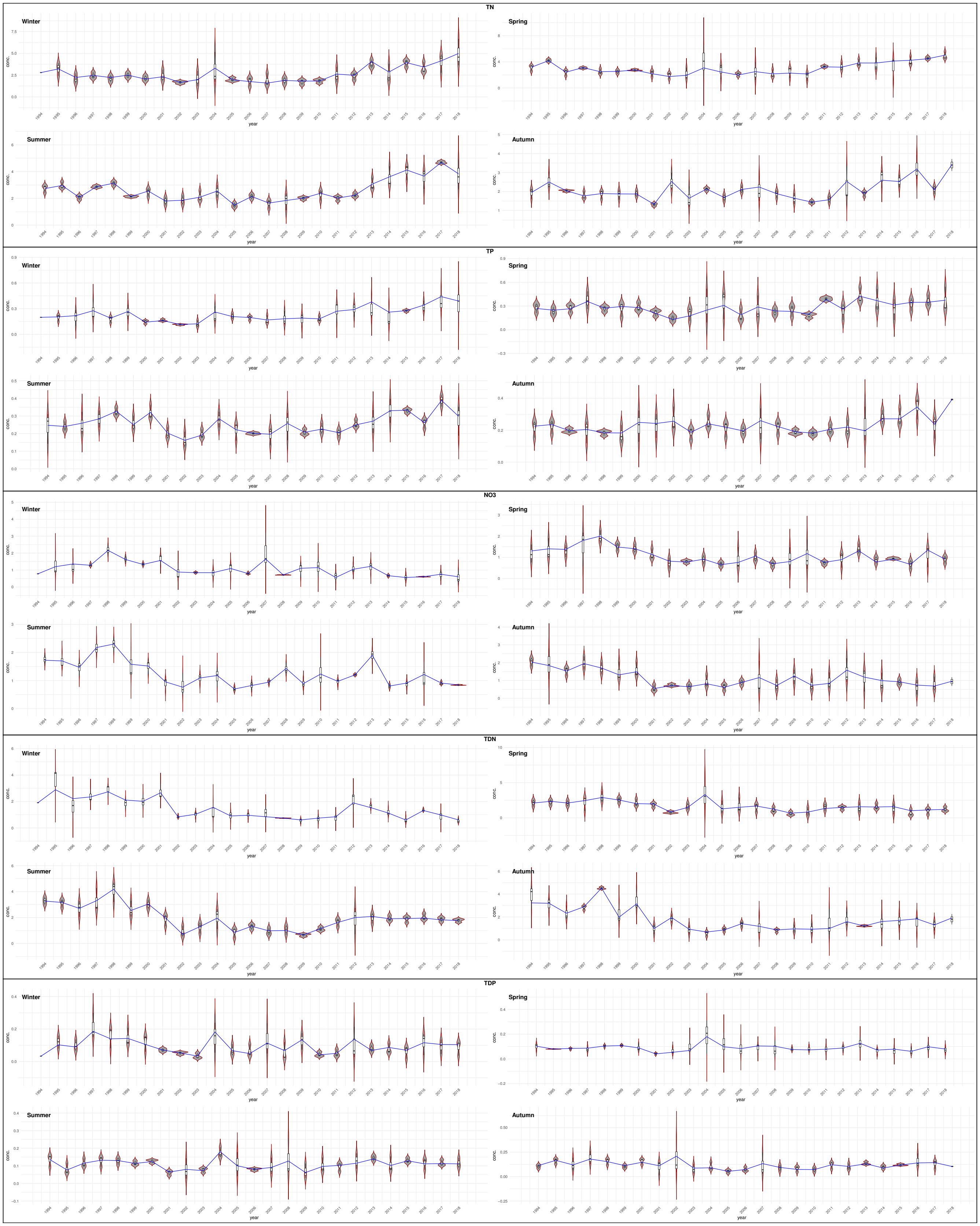

Figure 4: **Annual trend violin plots.** Violin plots for monthly averaged data distribution in streams across contiguous US for each year with the mean values connected by a blue line

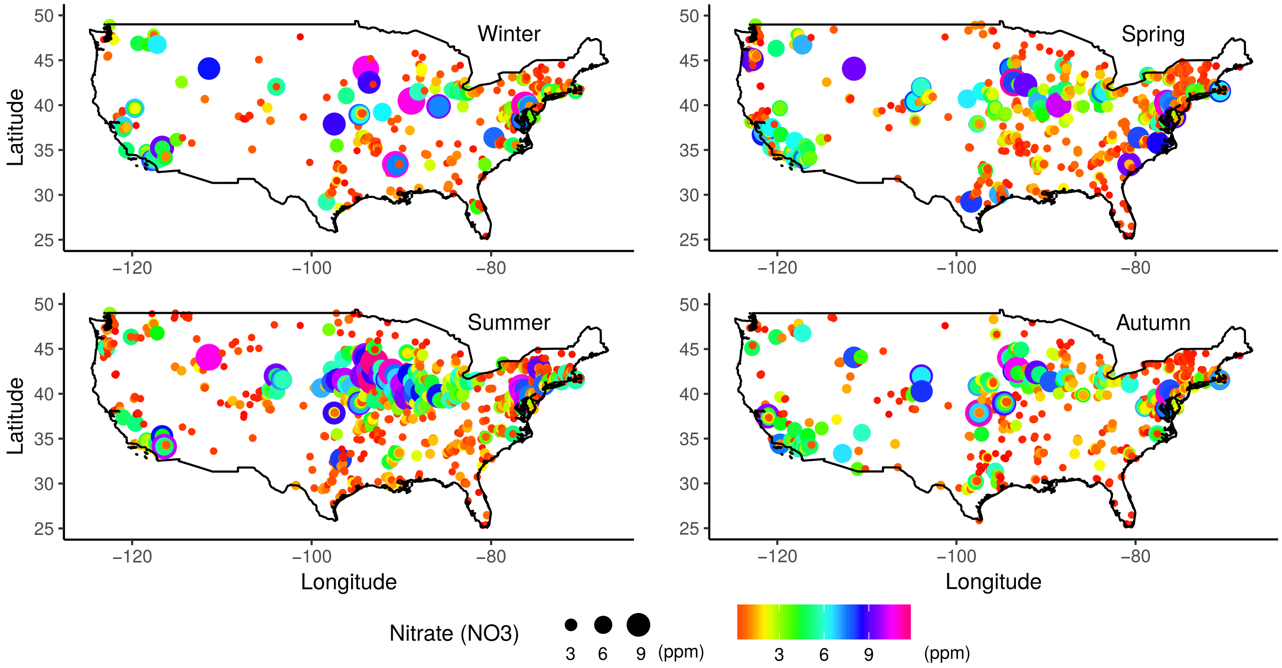

Figure 5: **Distribution map for NO3.** Spatial and seasonal distribution of the Water Quality Portal's stations. The Nitrate (NO3) seasonal mean for each station is labelled by a colour circle which also increase in size in accordance to the value NO3 values.

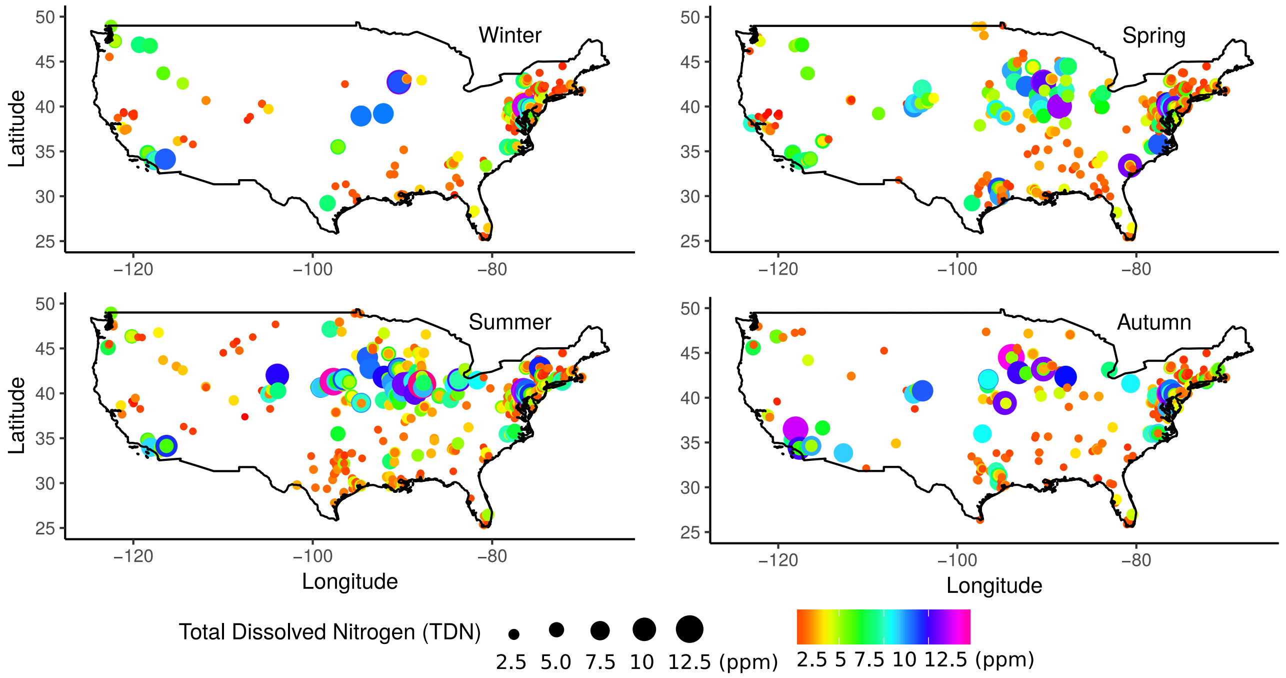

Figure 6: **Distribution map for TDN.** Spatial and seasonal distribution of the Water Quality Portal's stations. The Total Dissolved Nitrogen (TDN) seasonal mean for each station is labelled by a colour circle which also increase in size in accordance to the value TDN values.

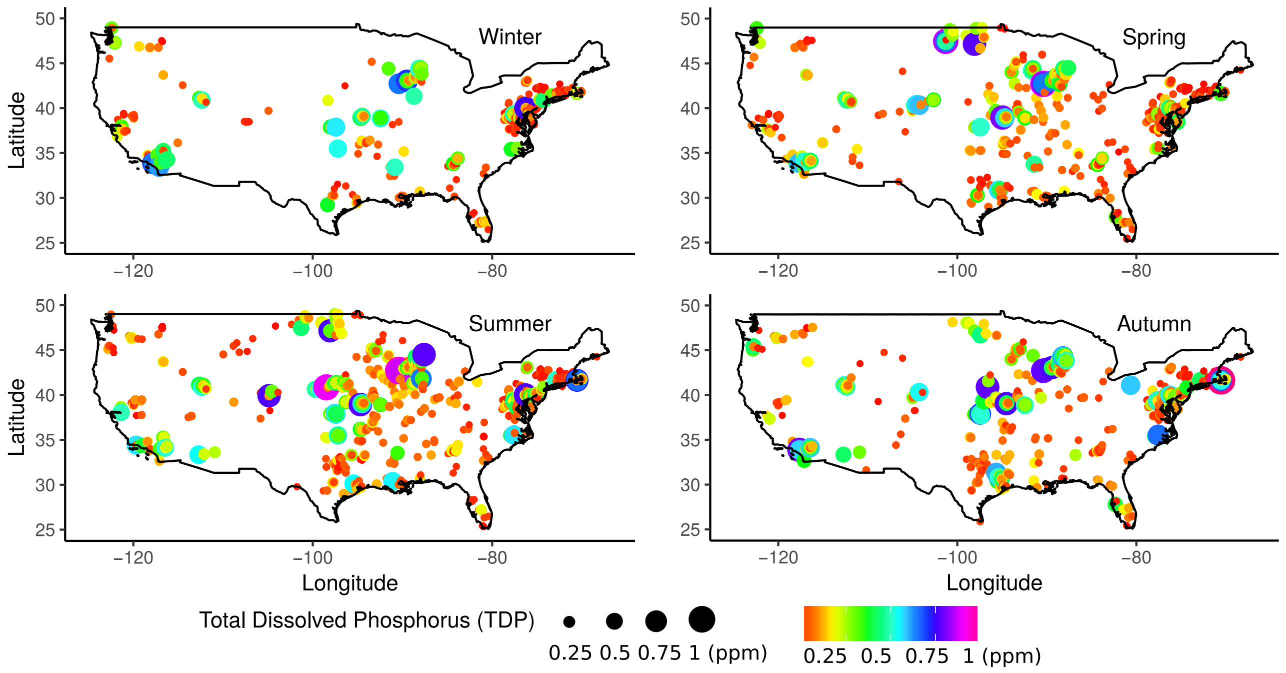

Figure 7: **Distribution map for TDP.** Spatial and seasonal distribution of the Water Quality Portal's stations. The Total Dissolved Phosphorus (TDP) seasonal mean for each station is labelled by a colour circle which also increase in size in accordance to the value TP values.

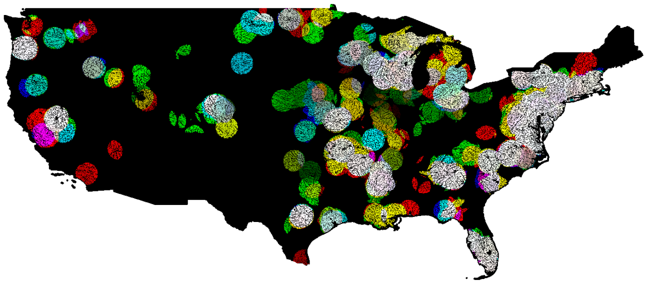

(a) a. TN

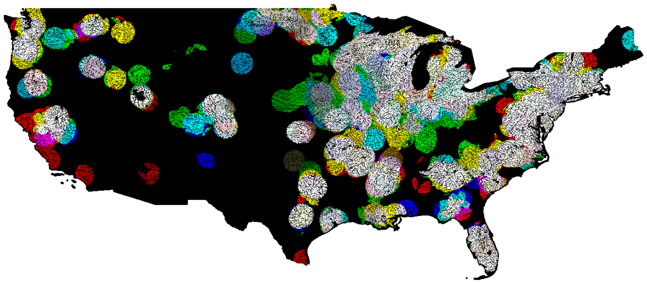

(b) b. TP

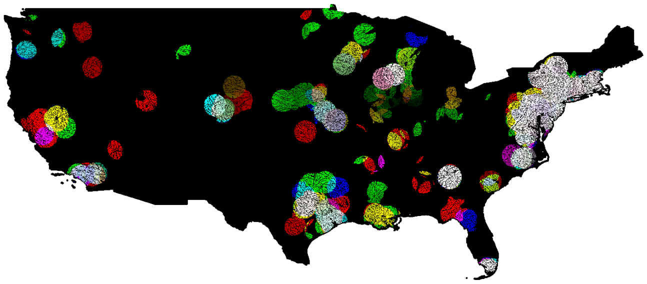

(c) c. TDN

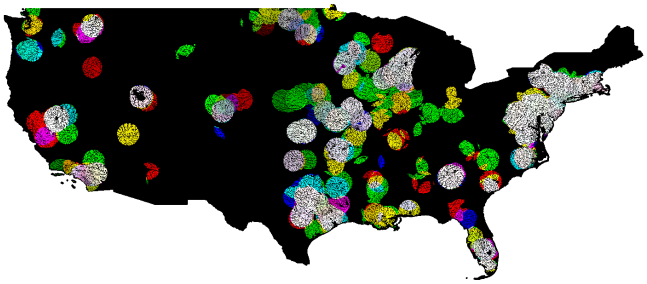

(d) d. TDP

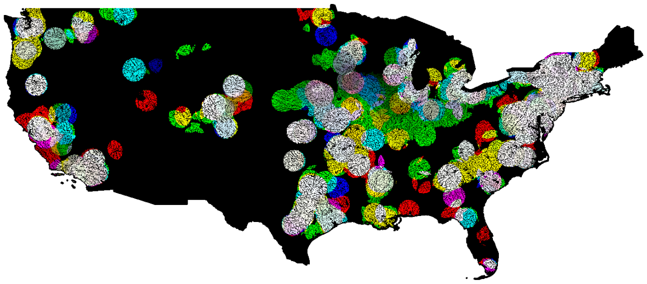

(e) e. NO3

Figure 8: **Seasonal variation RGB maps**. Seasonable difference plots using Winter as the reference. Observation data were first interpolated by averaging among neighbours within the distance of 1 degree and then normalised by the sum of all pixel values for each season. Afterwards, absolute differences were taken between non-Winter and Winter season by season to produce three difference maps. The values of difference maps were scaled between 0 and 255 first and followed by inversion to produce the final overlaied three-band RGB maps. Whiter areas indicates closer in similarity among the four seasons.

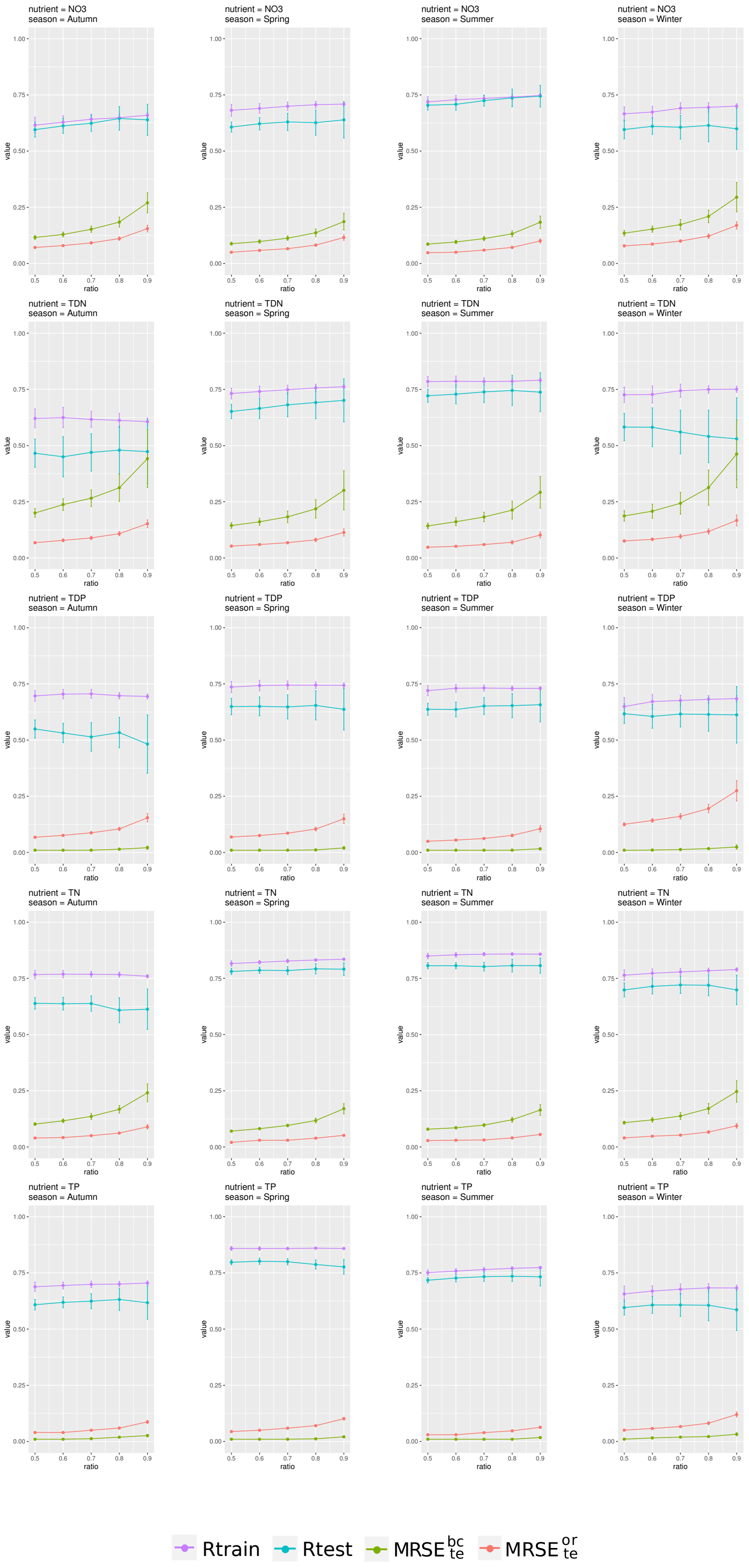

Figure 9: **Splitting variations.** Scatter plots for various splitting scenarios for each chemical nutrient. The horizontal axis represents a series of post-split proportions of the training set out of all the data. The legends Rtrain represents the Pearson Coefficient for the training set, Rtest for the testing set, MRSE_{tr}^{bc} for the training set, MRSE_{te}^{bc} for the testing set, MRSE_{te}^{or} for the testing set after a reverse Box-Cox transfer that converted the predicted values back to their original concentrations in the unit of ppm. $\mathrm{MRSE} = \sqrt{\sum_i^n (x_i - \hat{x}_i)^2/n}$, where x_i represents the observation and $\hat{x}_i$ represents the predicted value for data i.

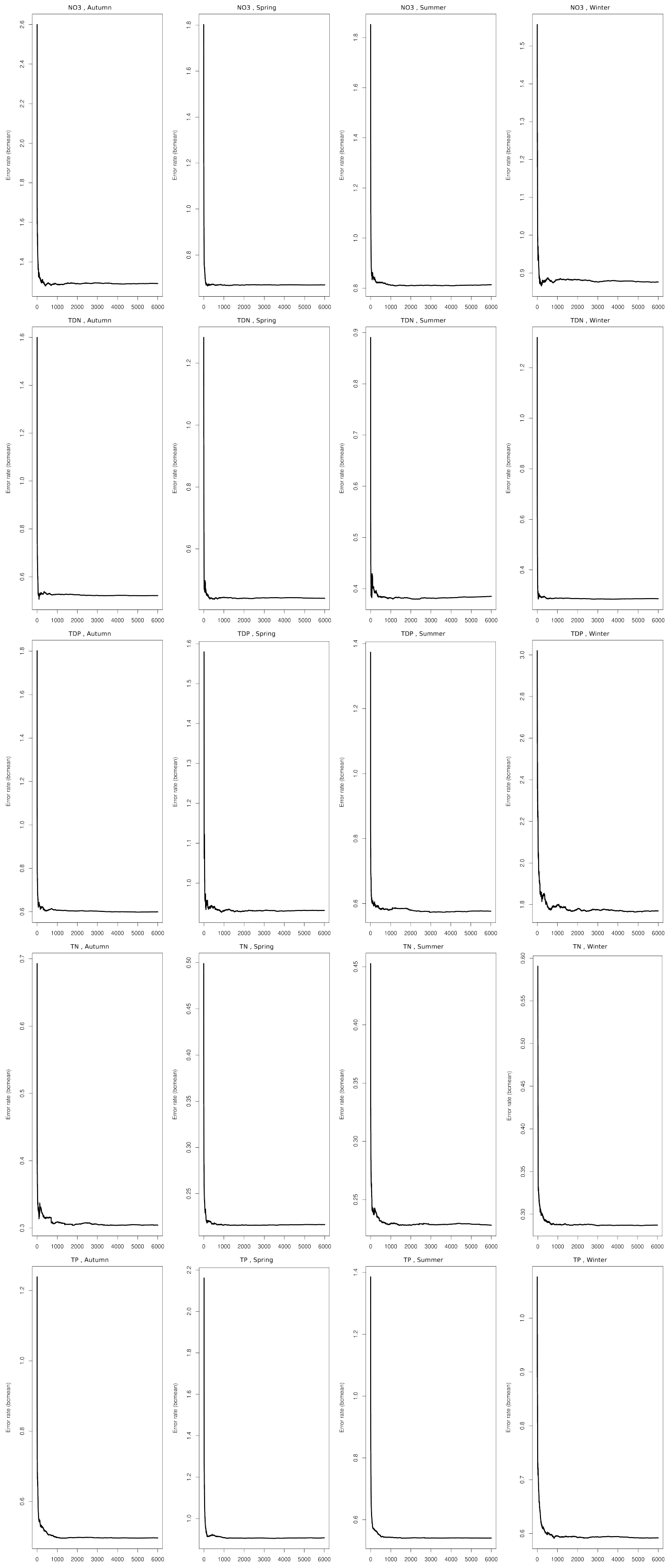

Figure 10: **Plots for model tuning.** Connected scatter plots of model errors vs. number of trees.

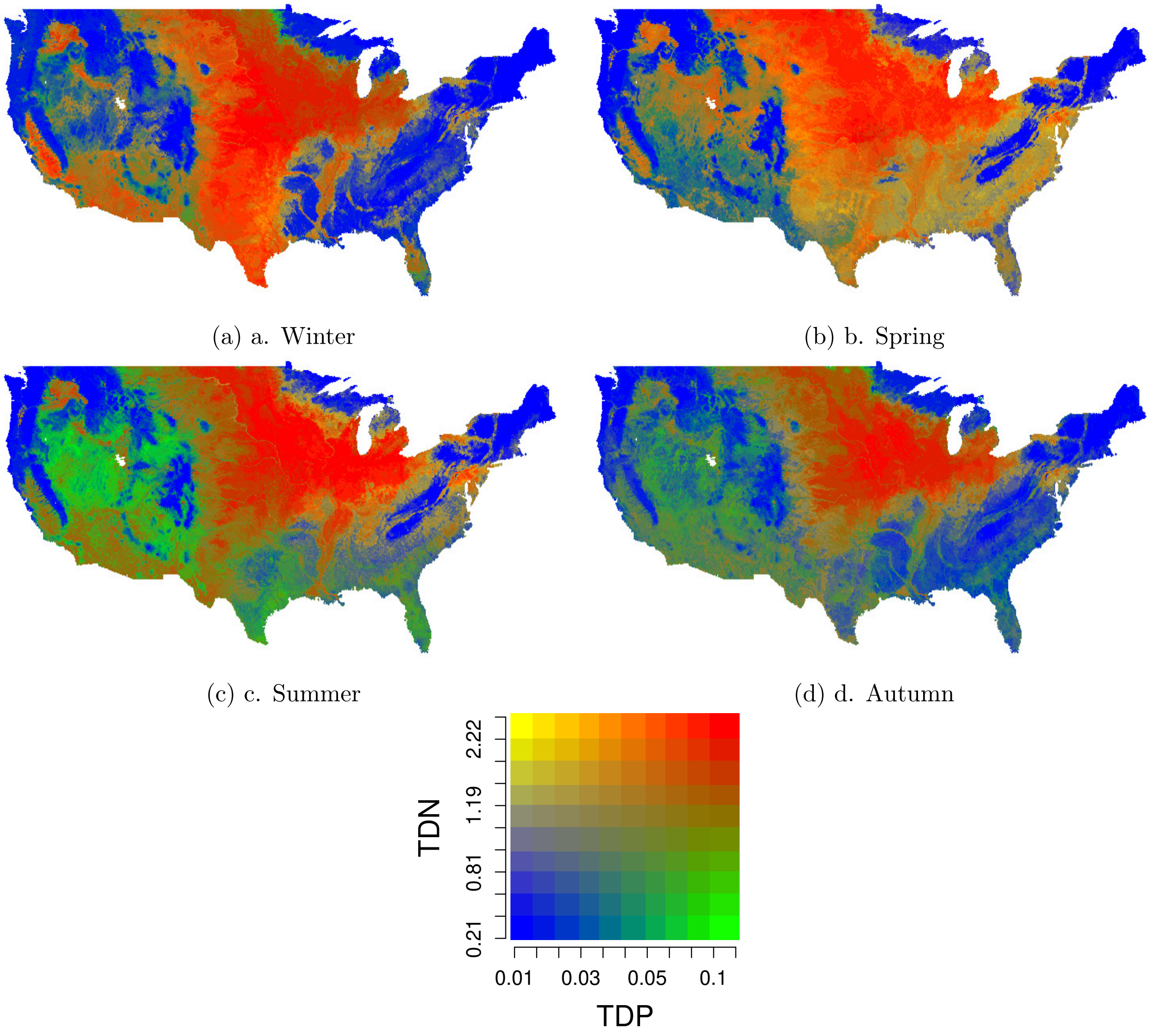

(a) a. Winter

(b) b. Spring

(c) c. Summer

(d) d. Autumn

Figure 11: **Bivariate maps for TDN and TDP.** Bivariate maps showing the predicted total dissolved nitrogen (TDN) and total dissolved phosphorus (TDP) values in ppm across the four seasons.

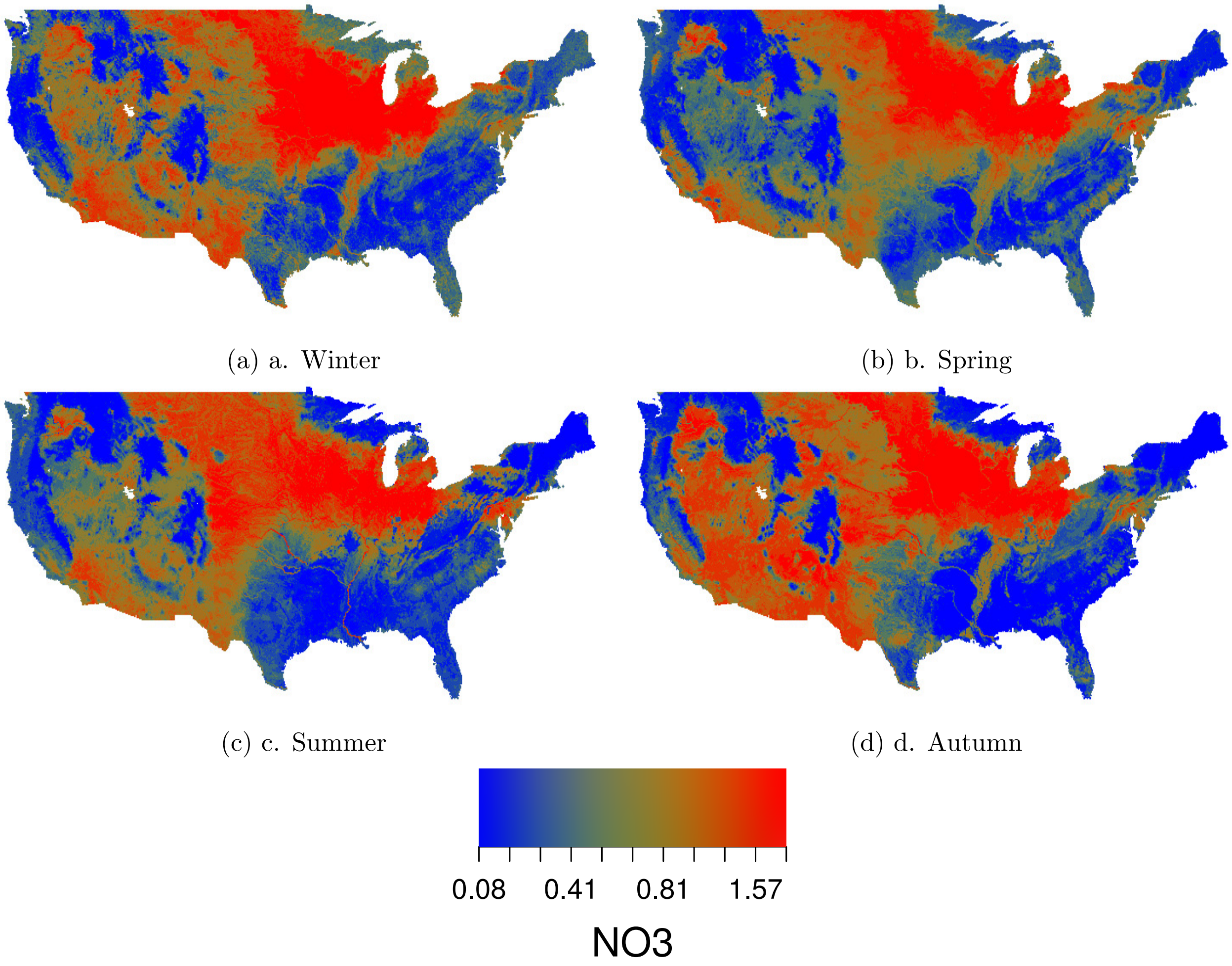

(a) a. Winter

(b) b. Spring

(c) c. Summer

(d) d. Autumn

Figure 12: **Seasonal prediction maps for NO3**Maps showing the predicted Nitrate (NO3) values in ppm across the four seasons.

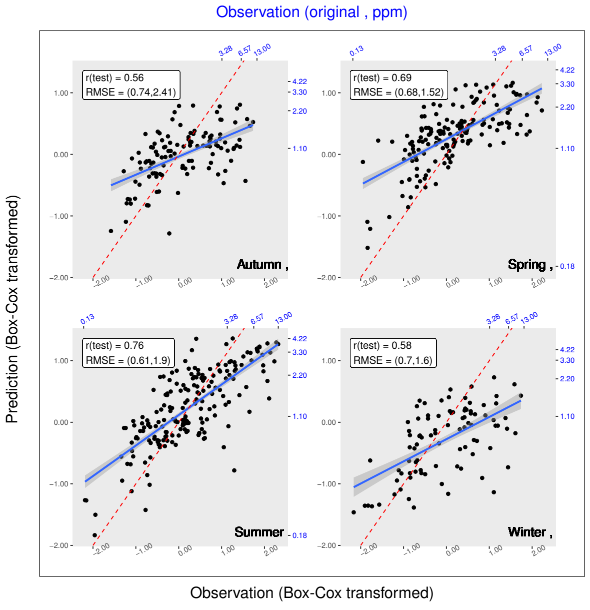

(a) TDN

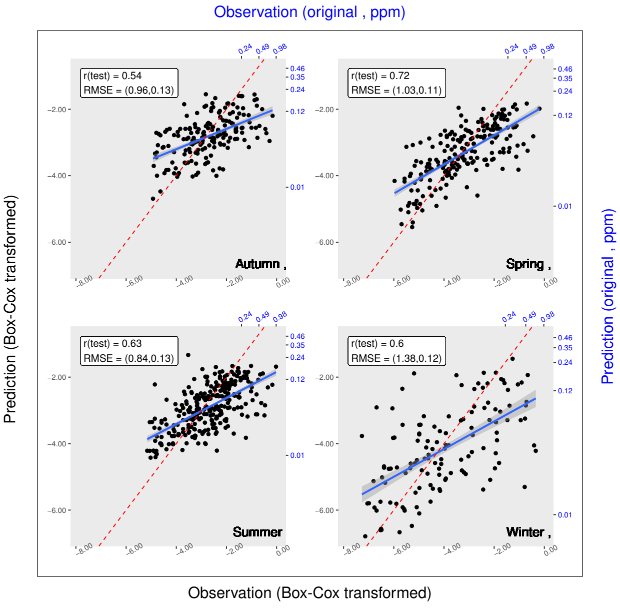

(b) TDP

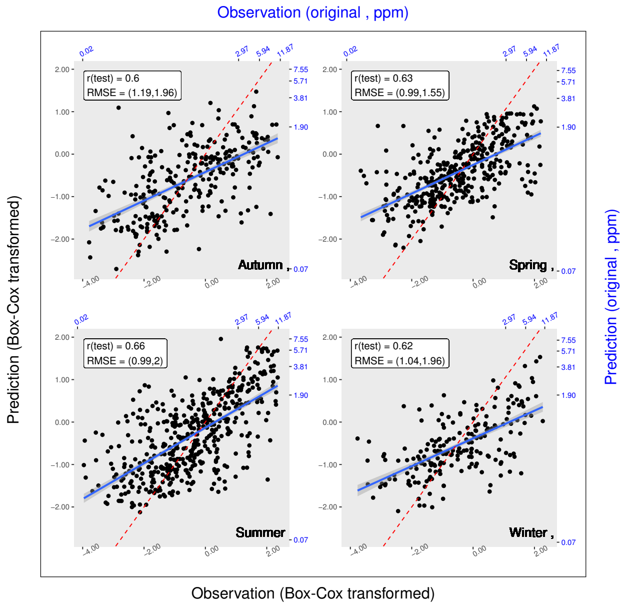

(c) NO3

Figure 13: **Correlation plots for TDN, TDP and NO3 in testing.** Seasonal correlation plots for TDN, TDP and NO3 for the testing data set. Horizontal axes represent the observations and vertical axes represent the predicted values. Ticks labelled in black are box-cox transformed values and ticks in blue are original values in ppm. Pearson coefficients (r) and RMSE are given in the upper-left corner box. Two values in parenthesis followed after RMSE were calculated based on box-cox transformed values and the original observation values respectively.

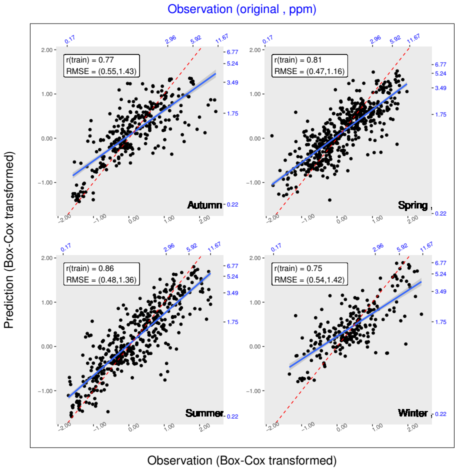

(a) TN

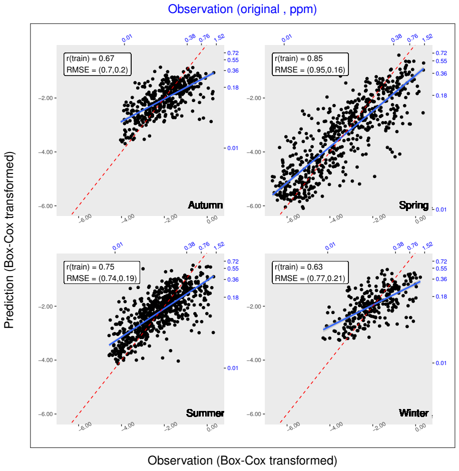

(b) TP

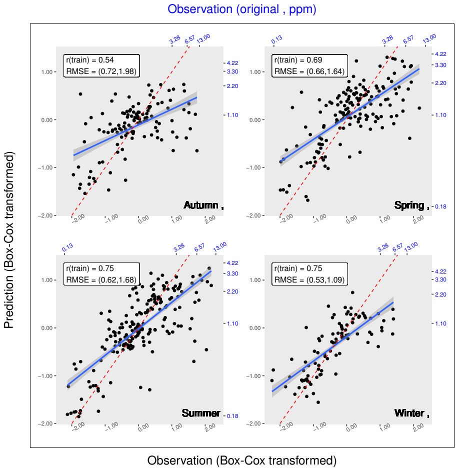

(c) TDN

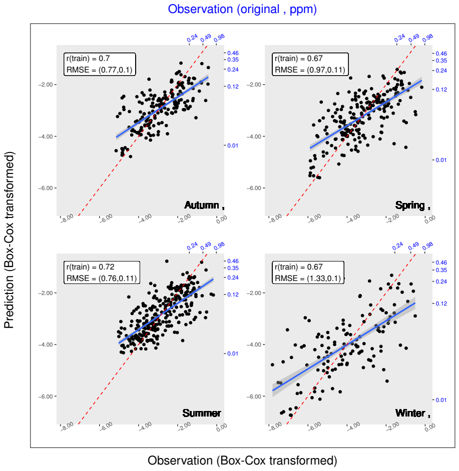

(d) TDP

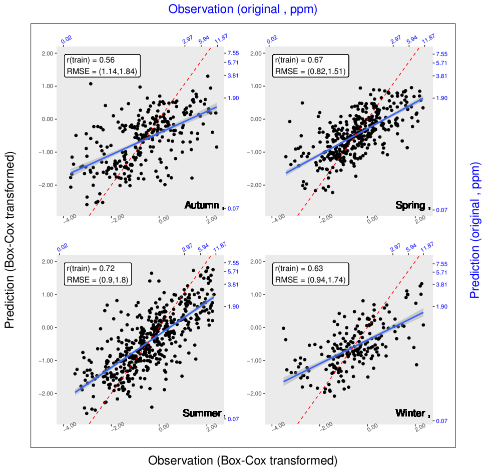

(e) NO3

Figure 14: **Correlation plots for all chemical species in training.** Seasonal correlation plots for each chemical species for the training data set. Horizontal axes represent the observations and vertical axes represent the predicted values. Ticks labelled in black are box-cox transformed values and ticks in blue are original values in ppm. Pearson coefficients (r) are given in the upper-left corner box.

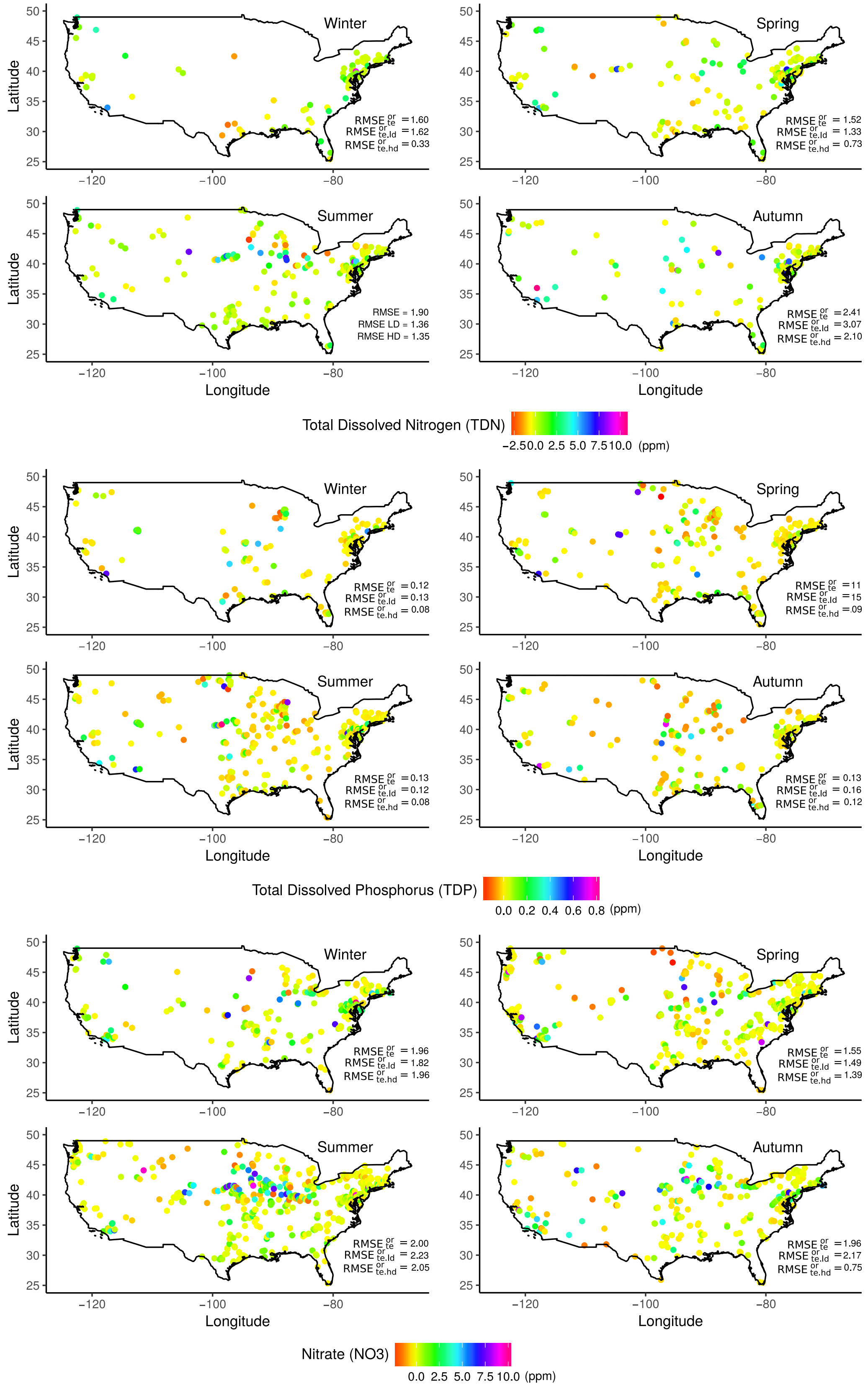

Figure 15: **Residual maps for TDN, TDP and NO3.** Residuals are computed using the testing sub-dataset (observations minus predictions). In each maps is also reported the RMSE$^{or}_{te}$ for the testing sub-daset in ppm, RMSE$^{or}_{te.ld}$ and RMSE$^{or}_{te.hd}$ using observation in the low/high density, respectively.

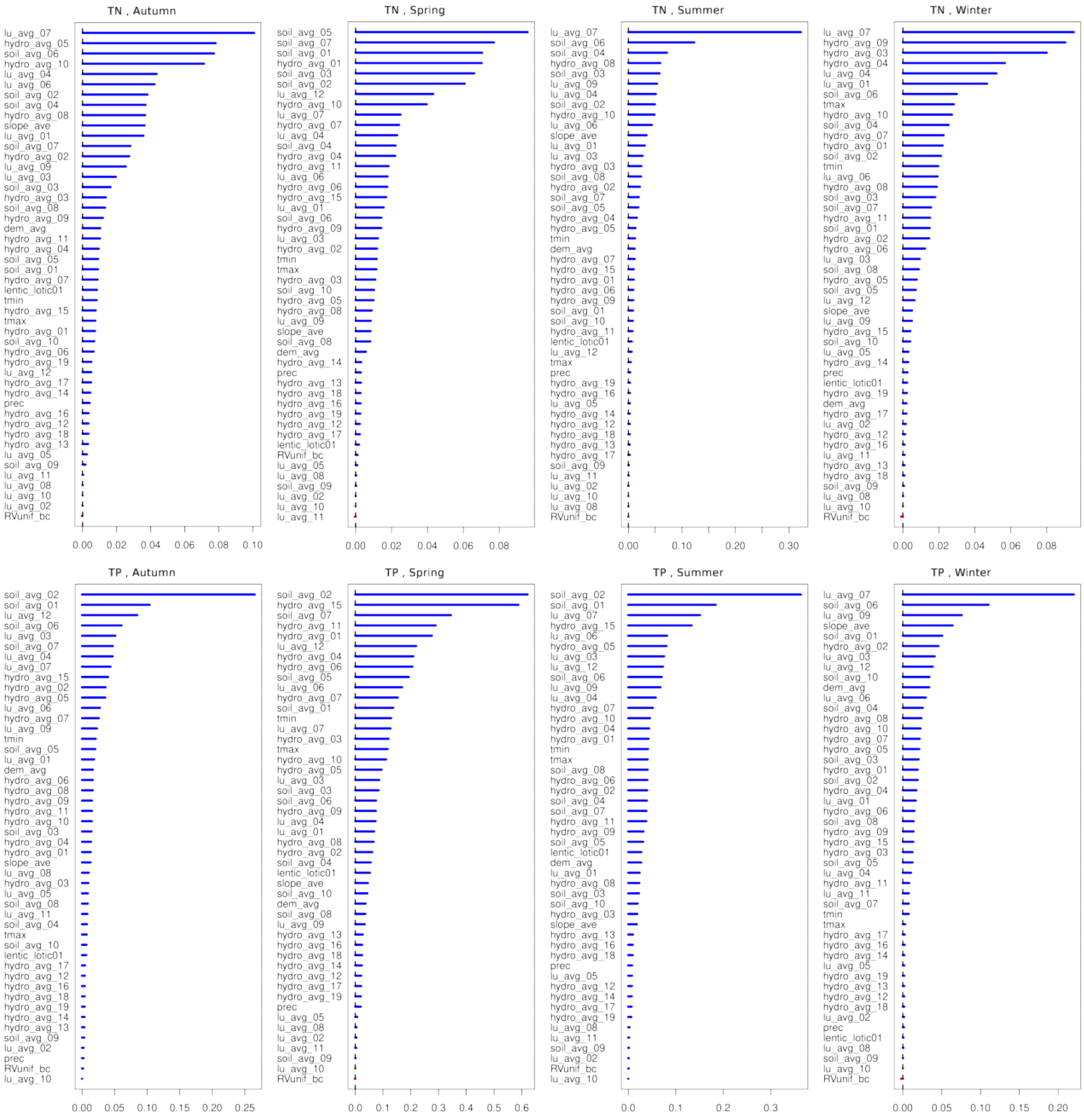

Figure 16: **Variable importance plots for TN and TP.**

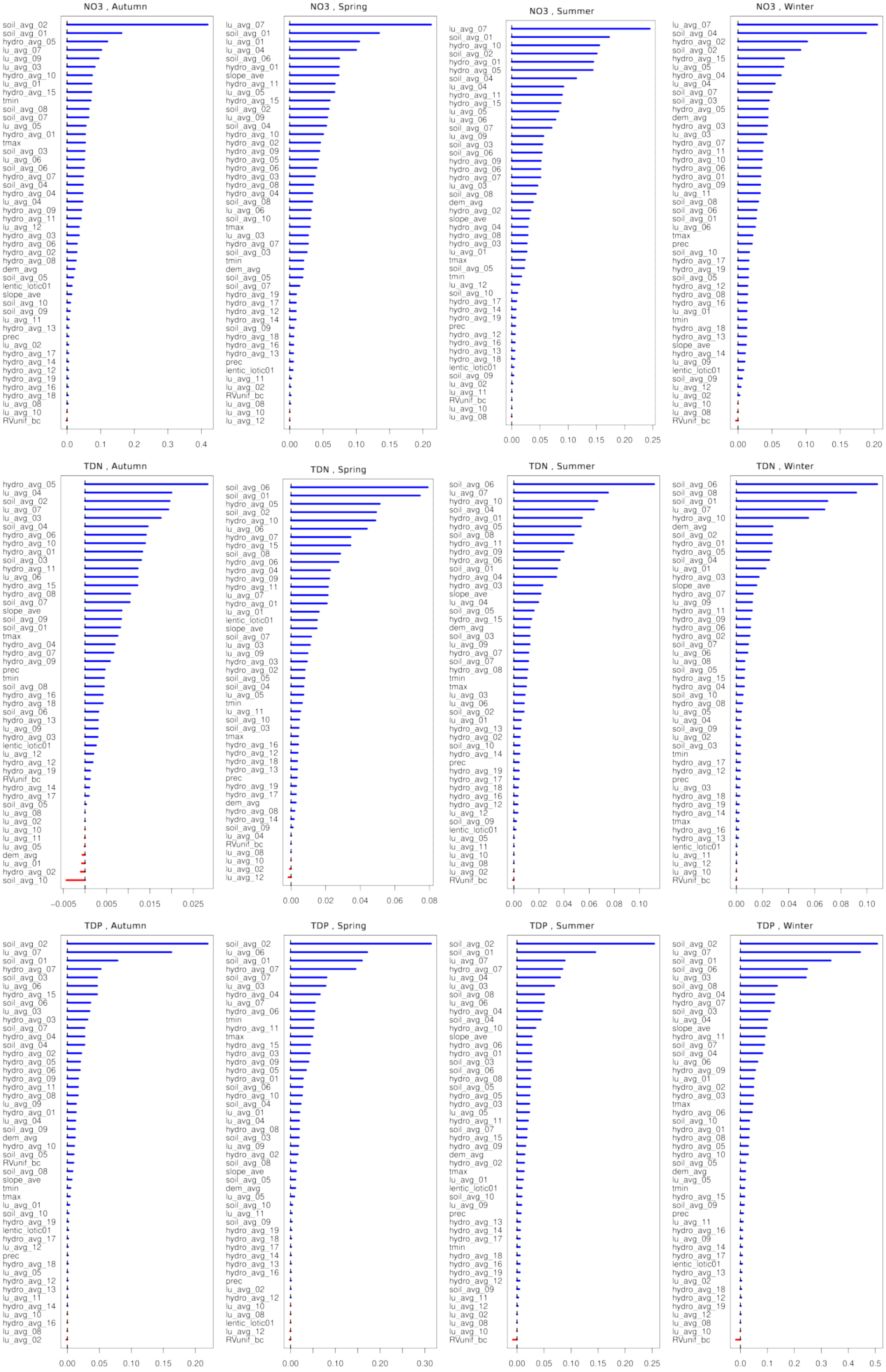

Figure 17: **Variable importance plots for TDN, TDP and NO3.**
